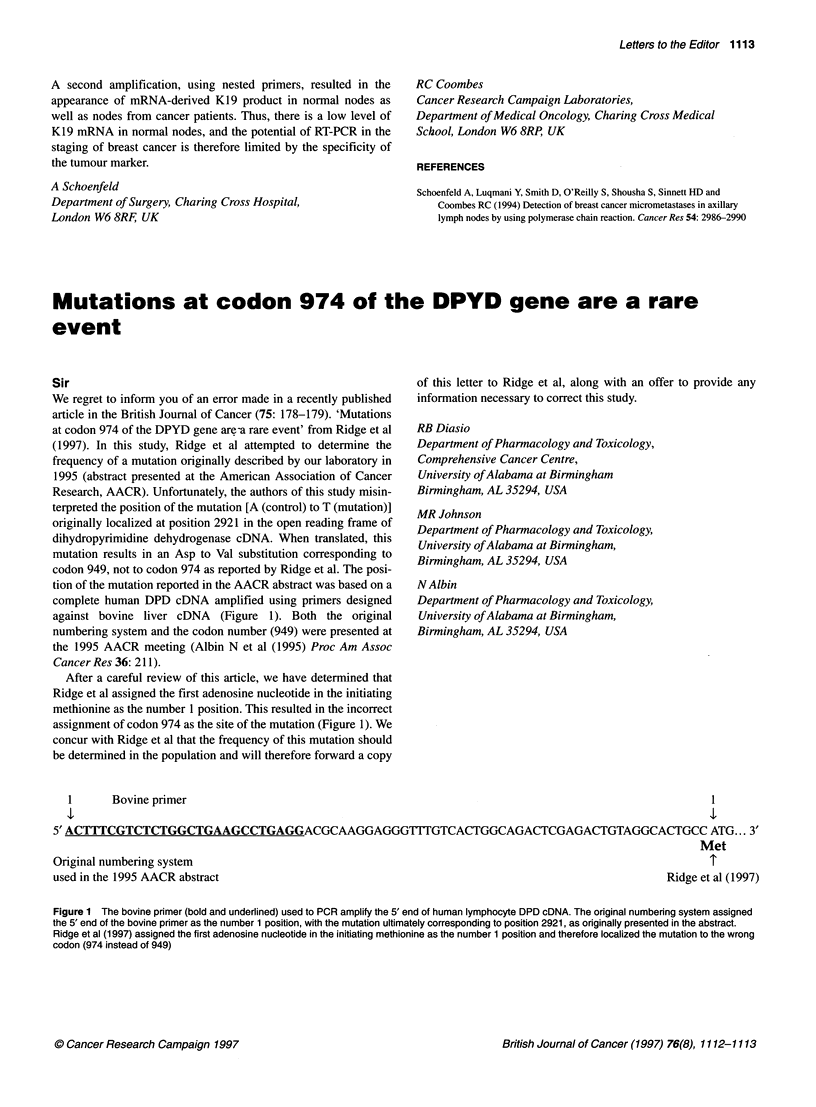# Mutations at codon 974 of the DPYD gene are a rare event.

**DOI:** 10.1038/bjc.1997.518

**Published:** 1997

**Authors:** R. B. Diasio, M. R. Johnson, N. Albin


### Mutations at codon 974 of the DPYD gene are a rare event

**Sir**

We regret to inform you of an error made in a recently published article in the British Journal of Cancer (**75**: 178–179). 'Mutations at codon 974 of the DPYD gene are a rare event' from Ridge et al (1997). In this study, Ridge et al attempted to determine the frequency of a mutation originally described by our laboratory in 1995 (abstract presented at the American Association of Cancer Research, AACR). Unfortunately, the authors of this study misinterpreted the position of the mutation [A (control) to T (mutation)] originally localized at position 2921 in the open reading frame of dihydropyrimidine dehydrogenase cDNA. When translated, this mutation results in an Asp to Val substitution corresponding to codon 949, not to codon 974 as reported by Ridge et al. The position of the mutation reported in the AACR abstract was based on a complete human DPD cDNA amplified using primers designed against bovine liver cDNA (Figure 1). Both the original numbering system and the codon number (949) were presented at the 1995 AACR meeting (Albin N et al (1995) *Proc Am Assoc Cancer Res* **36**: 211).

After a careful review of this article, we have determined that Ridge et al assigned the first adenosine nucleotide in the initiating methionine as the number 1 position. This resulted in the incorrect assignment of codon 974 as the site of the mutation (Figure 1). We concur with Ridge et al that the frequency of this mutation should be determined in the population and will therefore forward a copy of this letter to Ridge et al, along with an offer to provide any information necessary to correct this study.

*RB Diasio*
*Department of Pharmacology and Toxicology, Comprehensive Cancer Centre, University of Alabama at Birmingham Birmingham, AL 35294, USA*

*MR Johnson*
*Department of Pharmacology and Toxicology, University of Alabama at Birmingham, Birmingham, AL 35294, USA*

*N Albin*
*Department of Pharmacology and Toxicology, University of Alabama at Birmingham, Birmingham, AL 35294, USA*

1 Bovine primer ↓ ... 1 ↓

5′ **ACTTTCGTCTCTGGCTGAAGCCTGAGG**ACGCAAGGAGGGTTTGTCACTGGCAGACTCGAGACTGTAGGCACTGCC ATG... 3′

**Met** ↑

Original numbering system used in the 1995 AACR abstract — Ridge et al (1997)

**Figure 1** The bovine primer (bold and underlined) used to PCR amplify the 5′ end of human lymphocyte DPD cDNA. The original numbering system assigned the 5′ end of the bovine primer as the number 1 position, with the mutation ultimately corresponding to position 2921, as originally presented in the abstract. Ridge et al (1997) assigned the first adenosine nucleotide in the initiating methionine as the number 1 position and therefore localized the mutation to the wrong codon (974 instead of 949)

© Cancer Research Campaign 1997